# Role of Abdominal Aortic Balloon Placement in Planned Conservative Management of Placenta Previa With Placenta Increta or Percreta

**DOI:** 10.3389/fmed.2021.767748

**Published:** 2021-12-14

**Authors:** Ruihui Lu, Ran Chu, Qiannan Wang, Yintao Xu, Ying Zhao, Guowei Tao, Qi Li, Yuyan Ma

**Affiliations:** ^1^Department of Obstetrics and Gynecology, Qilu Hospital of Shandong University, Cheeloo College of Medicine, Shandong University, Jinan, China; ^2^Department of Radiology, Qilu Hospital of Shandong University, Jinan, China

**Keywords:** balloon placement in the abdominal aorta, placenta accreta spectrum, cesarean delivery, planned conservative management, adverse maternal event

## Abstract

**Background:** We investigated the role of balloon placement in the abdominal aorta (BPAA) in planned conservative management of placenta previa with placenta increta or percreta and the effects of BPAA on perinatal adverse maternal events.

**Methods:** This retrospective case-control study included women with placenta previa (increta or percreta), who underwent pregnancy termination at the Qilu Hospital of Shandong University between January 2016 and June 2019. Patients were categorized into the BPAA and non-BPAA groups based on the BPAA placement before delivery. The Chi-square and non-parametric rank-sum tests were used for the intergroup comparison of patient characteristics. The propensity score matching algorithm was used to minimize the intergroup differences in clinical characteristics. Logistic regression analysis was used to identify the factors associated with a high risk of adverse pregnancy outcomes. The area under the receiver operating characteristic curve [area under the curve (AUC)] was used to evaluate the classification of the selected high-risk factors.

**Results:** The study included 260 patients, and 104 patients were identified after propensity score matching. In the post-matched cohort, intraoperative blood loss was significantly lower in the BPAA than in the non-BPAA group (median 1,000 vs. 2,250 ml, *P* < 0.001). Intraoperative B-Lynch suture was performed in fewer patients in the BPAA (15.4 vs. 34.6%, *P* = 0.024) than in the non-BPAA group. The packed red blood cell (PRBC) transfusion rate was lower in the BPAA group (median 4 vs. 8 units, *P* < 0.001). Overall, 46 (45.1%) patients developed adverse maternal events; however, the rate of adverse maternal events was lower in the BPAA group (19.6 vs. 80.4%, *P* < 0.001). No ligation of the ascending branch of the uterine artery (*P* = 0.034), no BPAA (*P* < 0.001), intraplacental vascular lacunae (*P* = 0.046), and cervical hypervascularity (*P* = 0.001) were associated with a high risk of adverse perinatal maternal events. The AUC of the high-risk factors was 0.89 in the post-matched and 0.76 in the pre-matched cohorts.

**Conclusion:** Planned conservative management using BPAA significantly minimized the intraoperative blood loss, the need for a B-Lynch suture, and PRBC transfusion in patients with severe placenta accreta spectrum and placenta previa.

## Introduction

The placenta accreta spectrum (PAS), which includes placenta accreta, placenta increta, and placenta percreta, is primarily considered an entirely iatrogenic condition (1, 2). The increasing rates of cesarean deliveries and other surgeries or intrauterine manipulation that injures the endometrium have resulted in an increase in the incidence of PAS and placenta previa (3). The current prevailing hypothesis attributes the aforementioned findings to the fact that failure of normal decidualization in the scar area may lead to abnormally deep placentation. The trophoblast and villous tissue may invade the deeper myometrial layers, such as the vasculature and the bladder or other adjacent pelvic organs (2). PAS associated with previa increases the risk of peripartum hysterectomy, maternal morbidity, and even mortality, all of which affect maternal outcomes globally (4, 5).

Cesarean hysterectomy is the primary approach utilized to prevent severe hemorrhage secondary to invasive placentation (6); however, this procedure results in loss of fertility (7). Conservative treatment strategies that include uterus-preserving techniques tend to significantly reduce the adverse effects of uterus removal (8). Effective control of intraoperative blood loss and safety of the mother and neonate during the perinatal period are essential for conservative treatment.

Interventional radiologic techniques, particularly balloon catheter occlusion and embolization are considered valuable adjuncts to control bleeding (9). Balloon placement in the abdominal aorta (BPAA) occludes most of the pelvic blood supply and thereby reduces the bleeding-induced morbidity and provides a clear surgical field to facilitate the operation (10). Recent studies have shown that BPAA is used for the conservative management of PAS (11–13). However, owing to the small sample size, a bias cannot be excluded, and the results of these studies should be interpreted with caution. Due to the effects of emergency surgery, BPAA before cesarean delivery may not completely depend on the preference of the surgeon. The effectiveness of BPAA in patients with PAS may be affected by the patient's condition and clinical management strategies; therefore, well-designed case-control studies should be performed to confirm the safety and efficacy of BPAA.

In this study, we investigated the role of BPAA in planned conservative management of PAS in patients with placenta previa and the factors associated with a high risk of adverse perinatal maternal events.

## Materials and Methods

### General Information

This retrospective case-control study included 260 women with placenta previa (increta or percreta), who underwent pregnancy termination at the Qilu Hospital of Shandong University between January 2016 and June 2019. Inclusion criteria were as follows: (I) patient request for a conservative uterus-preserving therapeutic approach, (II) availability of prenatal obstetric ultrasonography records, (III) obstetrician-diagnosed placenta increta or percreta after cesarean delivery, and (IV) no history of coagulation disorders. Patients with placenta accreta rarely underwent BPAA before cesarean delivery and were, therefore, excluded from the study. The balloon catheter was inserted into the femoral artery and advanced to the bifurcation of the abdominal aorta before cesarean section in all patients who underwent BPAA, and the balloon was inflated with saline until the patient's pulse and oxygen saturation on the great toe could not be detected, then the surgeon can determine the optimal balloon volume (8–15 ml). Following delivery, the umbilical cord was clamped, and 0.9% sodium chloride solution was injected to inflate the balloon. Thereafter, we removed as much of the placenta as possible and used sutures including the B-Lynch suture to control bleeding. We repaired the uterus, and the catheters were removed after cesarean delivery. The continuous balloon inflation time duration is usually 10–30 min (with a 1–5 min interval, oxygen saturation on great toe returned to normal) during cesarean delivery.

### Patient Variables

The characteristics of patients were obtained from the medical records. Demographic and clinical variables included age at delivery, gestational history, obstetric complications, prenatal obstetric ultrasonography findings, serum hemoglobin (HGB) level, details regarding the cesarean delivery, and postoperative complications.

### Endpoints

Most patients underwent successful uterus-preserving conservative treatment except for those in whom cesarean hysterectomy was unavoidable. Therefore, diagnosis of PAS is primarily based on the placenta observed by the obstetrician during cesarean delivery. Placenta increta was defined as increased vascularity of the uterine serosa-bladder wall interface, myometrial thinning at the anterior uterine wall, and penetration of the placenta close to the serous layer. Placenta percreta was defined as placental penetration of the uterine surface with an invasion of the bladder or other adjacent organs (5). Currently, no standardized guidelines are available to define perinatal adverse events in patients with PAS. In the present study, we selected intraoperative blood loss ≥2,000 ml and transfused packed red blood cells (PRBC) ≥10 units as the criteria for adverse maternal events (7). All mothers and neonates underwent a 6-week follow-up after delivery.

### Statistical Analysis

Patients were categorized into non-BPAA and BPAA groups. Continuous and non-continuous data were compared using the Mann–Whitney *U*-test and the Chi-square test, respectively. The *p* < 0.05 was considered statistically significant.

We observed many characteristic differences (for example, ultrasonographic findings) between the non-BPAA and BPAA groups. We used a propensity score-matching algorithm to minimize the effect of these potential confounders on selection bias and maternal outcomes. Propensity scores were calculated for each patient using bivariate logistic regression analysis based on the following covariates: age, gravidity, parity, a history of dilation, and curettage of the uterus and cesarean delivery, serum HGB levels, gestational age, obstetric complications, placenta previa classification, prenatal ultrasonography results, and emergency cesarean delivery. The propensity scores were used to match patients in the non-BPAA group with those in the BPAA group at a fixed 1:1 ratio. We used the nearest available Mahalanobis metric matching within calipers, defined by the propensity score (caliper = 0.2).

Univariable logistic regression analysis was used to identify the clinical characteristics associated with adverse maternal events. Variables with *P* < 0.10 on the univariable logistic regression analysis were subjected to multivariate logistic regression analysis to confirm the independent high-risk factors. The results are presented using odds ratios with 95% CIs, and *p* values. Receiver operating characteristic (ROC) curves and the area under the curve (AUC) were used to evaluate the classification of independent high-risk factors. Factors with the AUC value closer to 1 were considered to have good discrimination power. The number of events per variable (EPV) was assumed to be at least 10 for the logistic regression to avoid relative bias.

All statistical analyses were performed using the IBM SPSS Statistics software, version 24.0. Propensity score matching, ROC, and AUC were calculated using the R software (version 3.6.2).

### Ethics

This retrospective study was approved by the Ethical Committee of Qilu Hospital of Shandong University (protocol number 2019013) with a waiver for informed consent. All patient data were anonymized to maintain the patients' privacy prior to the analysis.

## Results

### General Characteristics of Patients

**Figure 1:**
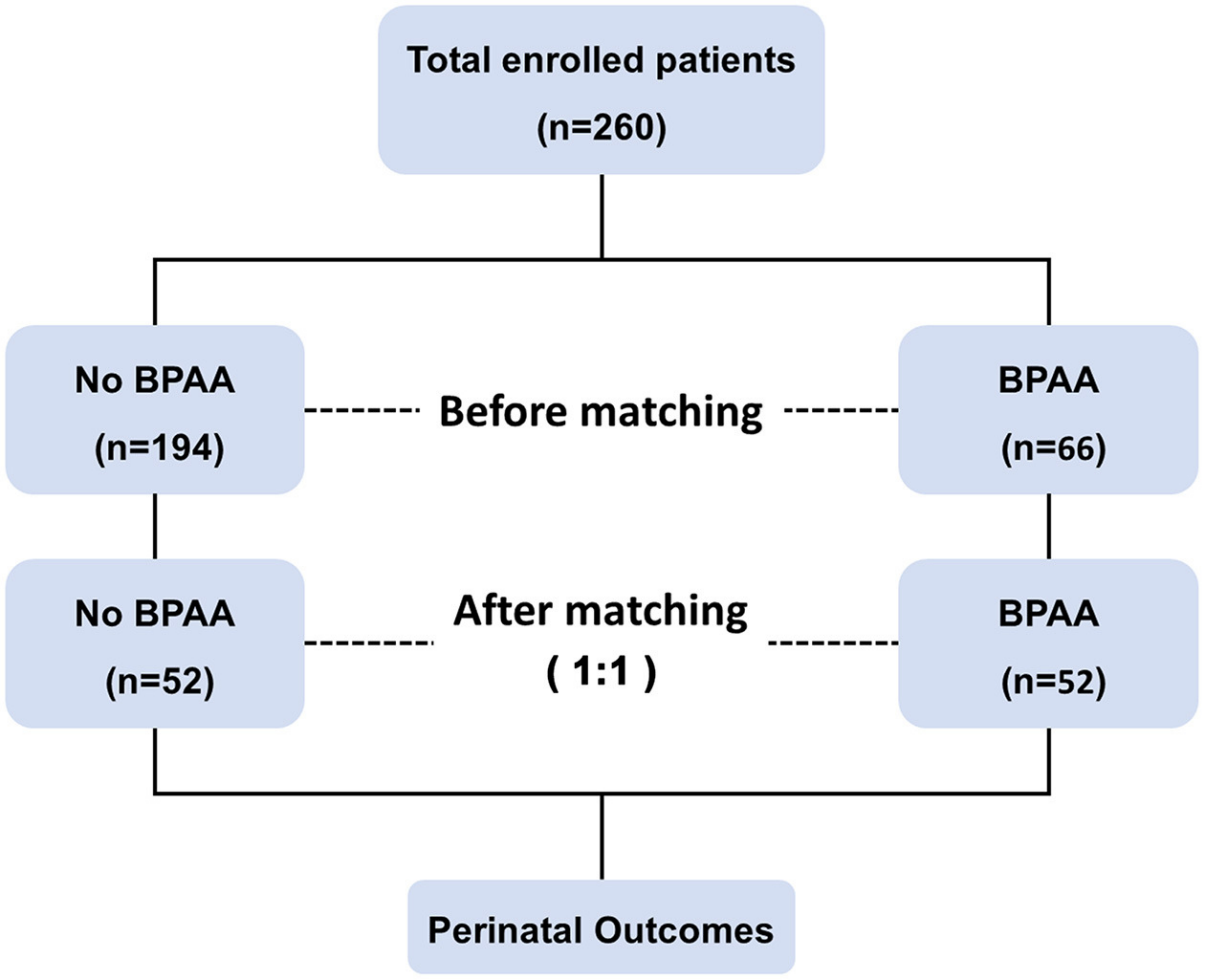
Flowchart of the study. BPAA, balloon placement of abdominal aorta.

Figure 1 shows the schema of the study. This study includes 260 pregnant women with placenta previa (placenta increta or percreta). Patients' median age at the time of delivery was 32 years (interquartile range 30–36 years), and the median gestational age was 255 days (interquartile range 245–260 days). All patients underwent cesarean delivery and pregnancy termination, with the birth of 171 (65.8%) full-term neonates. Adverse maternal events occurred in 106 (40.8%) women. The cohort was categorized into BPAA (BPAA performed before cesarean delivery) and non-BPAA groups; propensity score matching analysis yielded 104 patients (52 patients per group).

### Intergroup Comparison Before Propensity Score Matching

**Figure 2:**
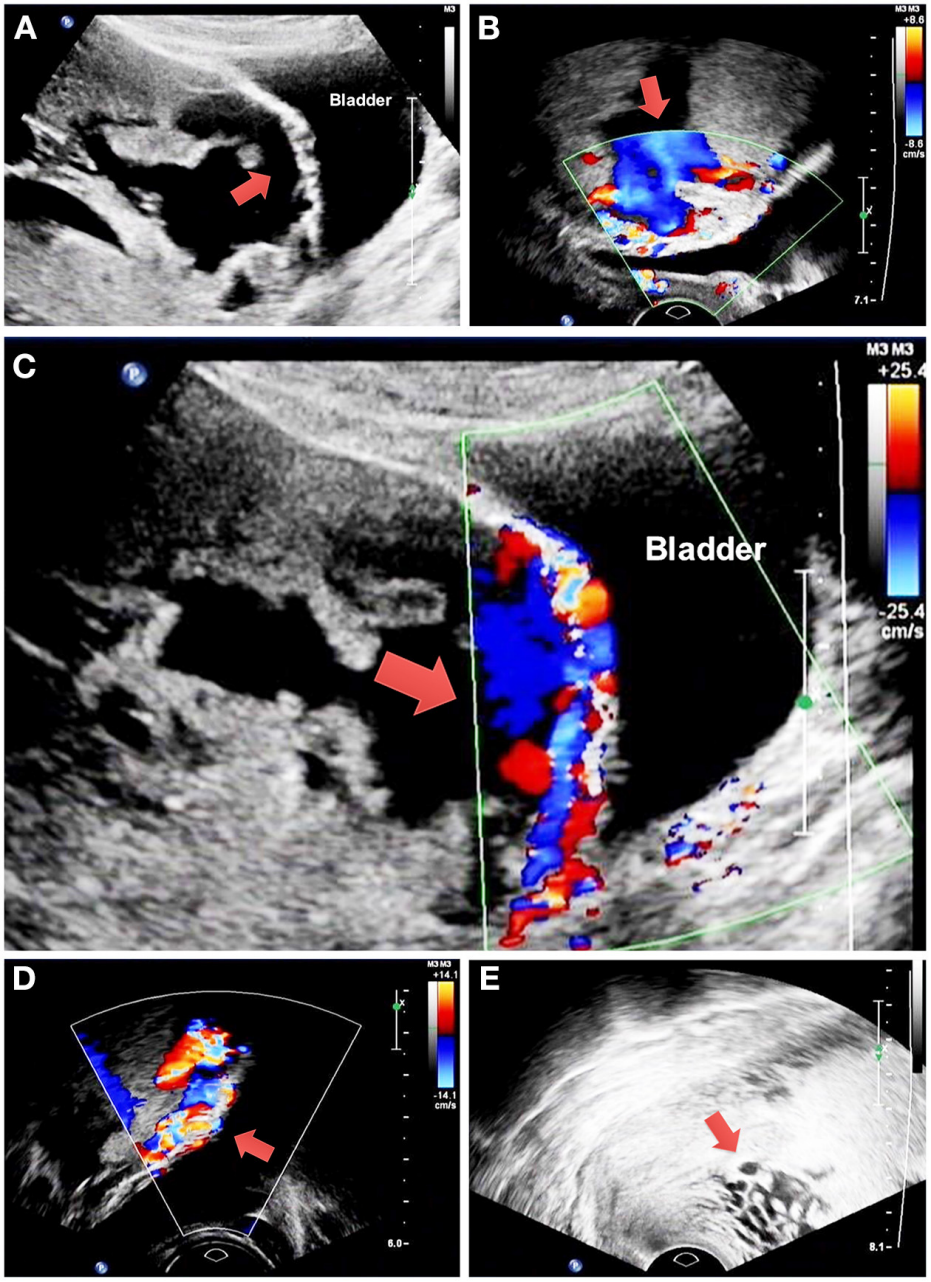
Doppler findings in patients with severe placenta accreta spectrum with previa. **(A)** The red arrow shows retroplacental myometrial thickness <1 mm and irregularity of the uterine-bladder interface. **(B)** The red arrow shows intraplacental vascular lacunae. **(C)** The red arrow shows hypervascularity of the uterine-placental margin and uterine serosa-bladder wall interface. **(D)** The red arrow shows hypervascularity of the uterine serosa-bladder wall interface. **(E)** The red arrow shows cervical hypervascularity. PAS, placenta accreta spectrum.

The study includes 194 and 66 patients in the non-BPAA and BPAA groups, respectively. Table 1 summarizes the characteristics of patients. Compared with patients in the non-BPAA group, a greater number of patients in the BPAA group showed abnormal placental vasculature on the preoperative ultrasonography, such as retroplacental myometrial thickness <1 mm (*P* = 0.002), intraplacental vascular lacunae (*P* < 0.001), hypervascularity of the uterine-placental margin (*P* = 0.090), irregularity of the uterine-bladder interface (*P* < 0.001), hypervascularity of the uterine serosa-bladder wall interface (*P* < 0.001), and the cervical hypervascularity (*P* = 0.011). We observed no significant intergroup differences in age, gestational age, pregnancy history, or other preoperative characteristics (*P* > 0.05). Figure 2 shows the Doppler characteristics of patients with severe PAS with previa.

**Table 1:** Characteristics before and after propensity score matching.

	**Before matching**			**After matching**		
**Characteristics**	**Non-BPAA (***n*** = 194)**	**BPAA (***n*** = 66)**	***p*** **value**	**Non-BPAA (***n*** = 52)**	**BPAA (***n*** = 52)**	***p*** **value**
**Age at delivery (years)**	32 (30–36)	33 (29–36)	0.507	33 (30–37)	32 (29–36)	0.540
**Gravidity**	3 (3–5)	3 (3–4)	0.334	3 (3–4)	3 (3–4)	0.997
**Parity**	1 (1–2)	1 (1–2)	0.838	1 (1–2)	1 (1–2)	0.603
**History of dilatation and curettage of uterine**	1 (0–2)	1 (0–2)	0.798	0 (0–1)	1 (0–1)	0.510
**Previous cesarean section**			0.093			0.658
≤ 1	143 (73.7)	49 (74.2)		37 (71.2)	39 (75.0)	
>1	51 (26.3)	17 (25.8)		15 (28.8)	13 (25.0)	
**Preoperative HGB level (g/L)**	105 (95–116)	105 (96–115)	0.801	106 (95–117)	105 (96–115)	0.868
**Gestational age (days)**	253 (243–260)	256 (249–261)	0.154	252 (243–260)	256 (247–261)	0.296
**Obstetric complications**						
Preeclampsia	5 (2.5)	1 (1.5)	1.000	2 (3.8)	1 (1.9)	1.000
Gestational diabetes mellitus	21 (10.8)	7 (10.6)	0.961	8 (15.4)	4 (7.7)	0.220
**Placenta previa classification**			0.093			0.390
Marginal	34 (17.5)	5 (7.6)		8 (15.4)	5 (9.6)	
Partial	6 (3.1)	1 (1.5)		1 (1.9)	0 (0.0)	
Complete	154 (79.4)	60 (90.9)		43 (82.7)	47 (90.4)	
**Prenatal ultrasound results**						
Retroplacental myometrial thickness <1 mm	126 (64.9)	56 (84.8)	0.002	38 (73.1)	42 (80.8)	0.352
Vascular lacunae within the placenta	99 (51.0)	56 (84.8)	<0.001	39 (75.0)	42 (80.8)	0.478
Hypervascularity of uterine-placental margin	125 (64.4)	50 (75.8)	0.090	37 (71.2)	41 (78.8)	0.365
Irregularity of uterine-bladder interface	29 (14.9)	30 (45.5)	<0.001	16 (30.8)	16 (30.8)	1.000
Hypervascularity of the uterine serosa-bladder wall interface	42 (21.6)	35 (53.0)	<0.001	23 (44.2)	21 (40.4)	0.691
Hypervascularity of cervix	18 (9.3)	14 (21.2)	0.011	7 (13.5)	7 (13.5)	1.000
**Emergency cesarean section**	25 (12.9)	3 (4.5)	0.059	7 (13.5)	3 (5.8)	0.183
**Type of PAS**			0.565			0.160
Placenta increta	178 (91.8)	62 (93.3)		45 (86.5)	50 (96.2)	
Placenta percreta	16 (8.2)	4 (6.1)		7 (13.5)	2 (3.8)	

*Values are median (interquartile range) or n (%). BPAA, balloon placement in the abdominal aorta; HGB, hemoglobin; PAS, placenta accrete spectrum*.

### Intergroup Comparison After Propensity Score Matching

**Figure 3:**
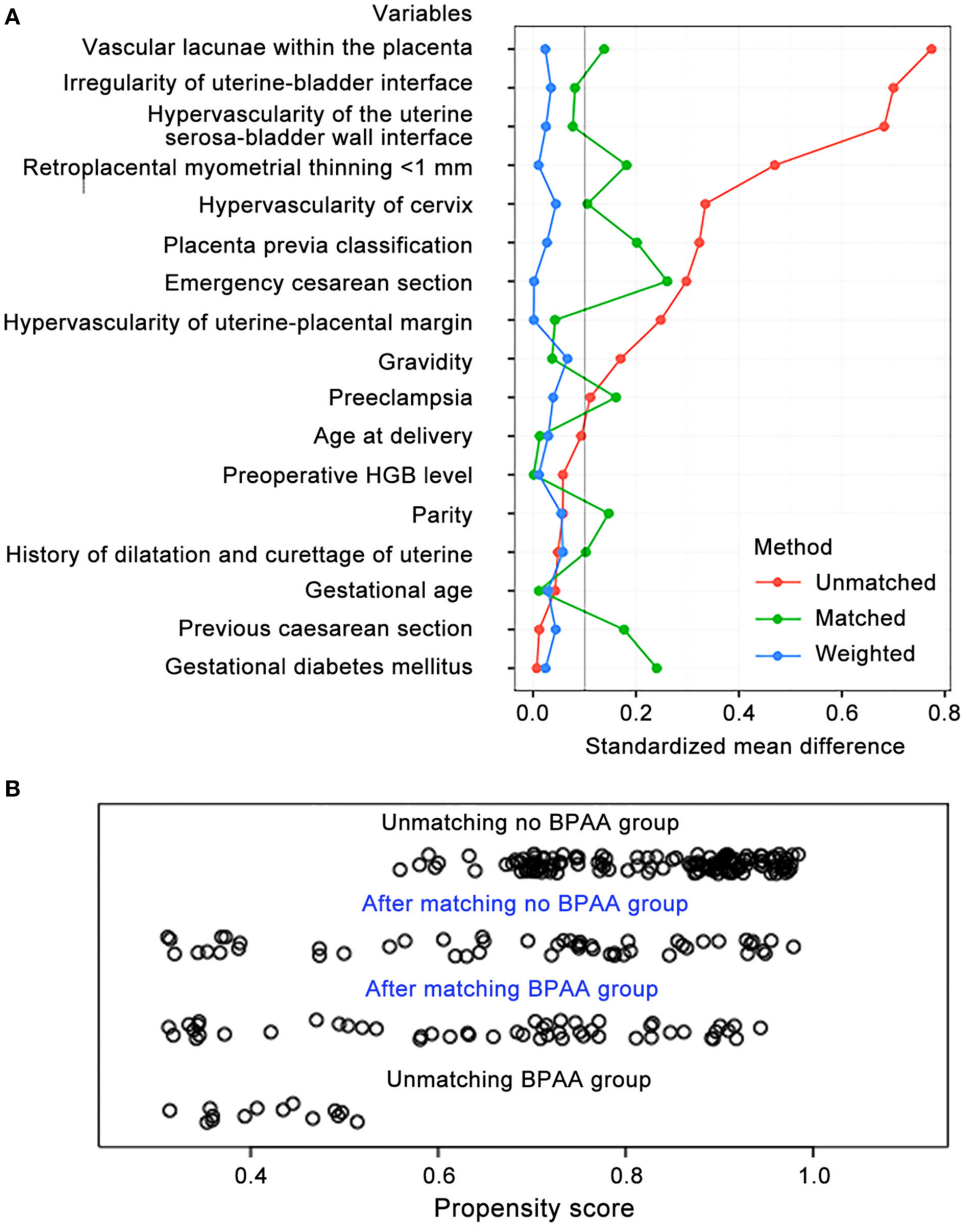
Propensity score matching of variables. **(A)** The standardized mean differences in variables between the non-BPAA and BPAA groups. **(B)** The distribution of the propensity scores before and after score-matching analysis. BPAA, balloon placement in the abdominal aorta; HGB, hemoglobin.

We observed bias between the non-BPAA and BPAA groups; therefore, the propensity score was calculated based on all clinical characteristics recorded before cesarean delivery. Using propensity score matching, 52 patients were included in each group. After matching, both groups included a lesser number of patients; however, we observed no significant intergroup differences in the characteristics of patients. Figure 3A shows that the standardized mean intergroup difference in variables was < 0.1, which indicates a negligible intergroup imbalance. Figure 3B shows the distribution of propensity scores before and after propensity matching of the cohort. Propensity score matching led to a uniform distribution of propensity scores in the cohort.

### Intergroup Comparison of Perinatal Outcomes Before and After Propensity Score Matching

Perinatal outcomes include surgical, neonatal, and adverse maternal events (Table 2). In the pre-matched cohort, the operation time (median 108 vs. 90 min, *P* = 0.026) and length of hospitalization (median 14 vs. 10 days, *P* < 0.001) were longer in the BPAA group. The B-Lynch suture was required in fewer patients (16.7 vs. 33.5%, *P* = 0.009), and the intraoperative blood loss was significantly lesser (median 1,000 vs. 1,800 ml, *P* = 0.001) in the BPAA than in the non-BPAA group. The risk of adverse maternal events was significantly lower (13.2 vs. 86.8%, *P* < 0.001) in the BPAA than in the non-BPAA group. No significant intergroup differences were observed in postoperative complications or neonatal outcomes.

**Table 2:** Comparisons of perinatal outcomes before and after propensity score matching.

			**Before matching**			**After matching**		
**Perinatal outcomes**			**Non-BPAA (***n*** = 194)**	**BPAA (***n*** = 66)**	***p*** **value**	**Non-BPAA (***n*** = 52)**	**BPAA (***n*** = 52)**	***p*** **value**
**Surgical outcomes**								
Total operation time (mins)			90 (72–120)	108 (80–127)	0.026	107 (83–138)	107 (81–127)	0.682
Length of hospital stay (days)			10 (8–16)	14 (10–23)	<0.001	10 (8–17)	14 (9–22)	0.184
Postoperative length of hospital stay (days)			5 (4–7)	5 (4–7)	0.772	6 (5–8)	5 (4–7)	0.008
Intraoperative blood loss (ml)			1,800 (800–3,000)	1,000 (600–1,800)	0.001	2,250 (1,500–4,000)	1,000 (600–1,675)	<0.001
Units of PRBC transfused			4 (4–10)	4 (4–8)	0.194	8 (4–14)	4 (2–6)	<0.001
B-Lynch suture			65 (33.5)	11 (16.7)	0.009	18 (34.6)	8 (15.4)	0.024
Ligation of ascending branch of uterine artery			33 (17.0)	14 (21.2)	0.444	12 (23.1)	10 (19.2)	0.631
Tourniquet binding the lower uterine segment			33 (17.0)	8 (12.1)	0.346	8 (15.4)	5 (9.6)	0.374
Hysterectomy			8 (4.1)	0 (0.0)	0.208	5 (9.6)	0 (0.0)	0.057
Bladder repair			16 (8.2)	4 (6.1)	0.565	7 (13.5)	2 (3.8)	0.160
Systemic infections			5 (2.6)	0 (0.0)	0.334	1 (1.9)	0 (0.0)	1.000
**Thrombotic complications**								
Pulmonary embolism			0 (0.0)	1 (1.5)	0.254	0 (0.0)	1 (1.9)	1.000
DVT or thrombotic requiring therapy			0 (0.0)	1 (1.5)	0.254	0 (0.0)	0 (0.0)	–
DIC			3 (1.5)	0 (0.0)	0.573	2 (3.8)	0 (0.0)	0.495
ICU			3 (1.5)	1 (1.5)	1.000	2 (3.8)	1 (1.9)	1.000
**Neonatal outcomes**								
Full term birth			130 (67.0)	41 (62.1)	0.470	37 (71.2)	32 (61.5)	0.299
Apgar score (point)	1 min	0–7	37 (19.1)	12 (18.2)	0.873	8 (15.4)	11 (21.2)	0.415
		8–10	157 (80.9)	54 (81.8)		44 (84.6)	41 (78.8)	
	5 min	0–7	20 (10.3)	8 (12.1)	0.682	3 (5.8)	8 (15.4)	0.364
		8–10	174 (89.7)	58 (87.9)		49 (94.2)	44 (84.6)	
Neonatal weight (g)			2,800 (2,400–3,200)	2,875 (2,484–3,163)	0.949	2,900 (2,400–3,238)	2,800 (2,458–3,100)	0.552
NICU			87 (44.8)	23 (34.8)	0.156	26 (50.0)	17 (32.7)	0.073
Death			13 (6.7)	6 (9.1)	0.584	2 (3.8)	6 (11.5)	0.269
**Adverse maternal event**			92 (86.8)	14 (13.2)	<0.001	37 (80.4)	9 (19.6)	<0.001

*Values are median (interquartile range) or n (%). PRBC, packed red blood cells; BPAA, balloon placement in the abdominal aorta; DVT, deep vein thrombosis; DIC, disseminated intravascular coagulation; ICU, intensive care unit; NICU, neonatal intensive care unit*.

In the post-matched cohort, the length of postoperative hospitalization (median 5 vs. 6 days, *P* = 0.008) was shorter and the PRBC transfusion rate (median 4 vs. 8 units, *P* < 0.001) was significantly lower in the BPAA group. No patient underwent hysterectomy in the BPAA group in contrast to five patients who underwent hysterectomy [0 (0.0%) vs. 5 (9.6%), *P* = 0.057] in the non-BPAA group. Similar to the findings in the pre-matched cohort, fewer patients required a B-Lynch suture (15.4 vs. 34.6%, *P* = 0.024), the intraoperative blood loss (median 1,000 vs. 2,250 ml, *P* < 0.001) was lesser, and the incidence of adverse maternal events (19.6 vs. 80.4%, *P* < 0.001) was lower in the BPAA group.

### Factors Associated With a High Risk of Adverse Maternal Events

**Figure 4:**
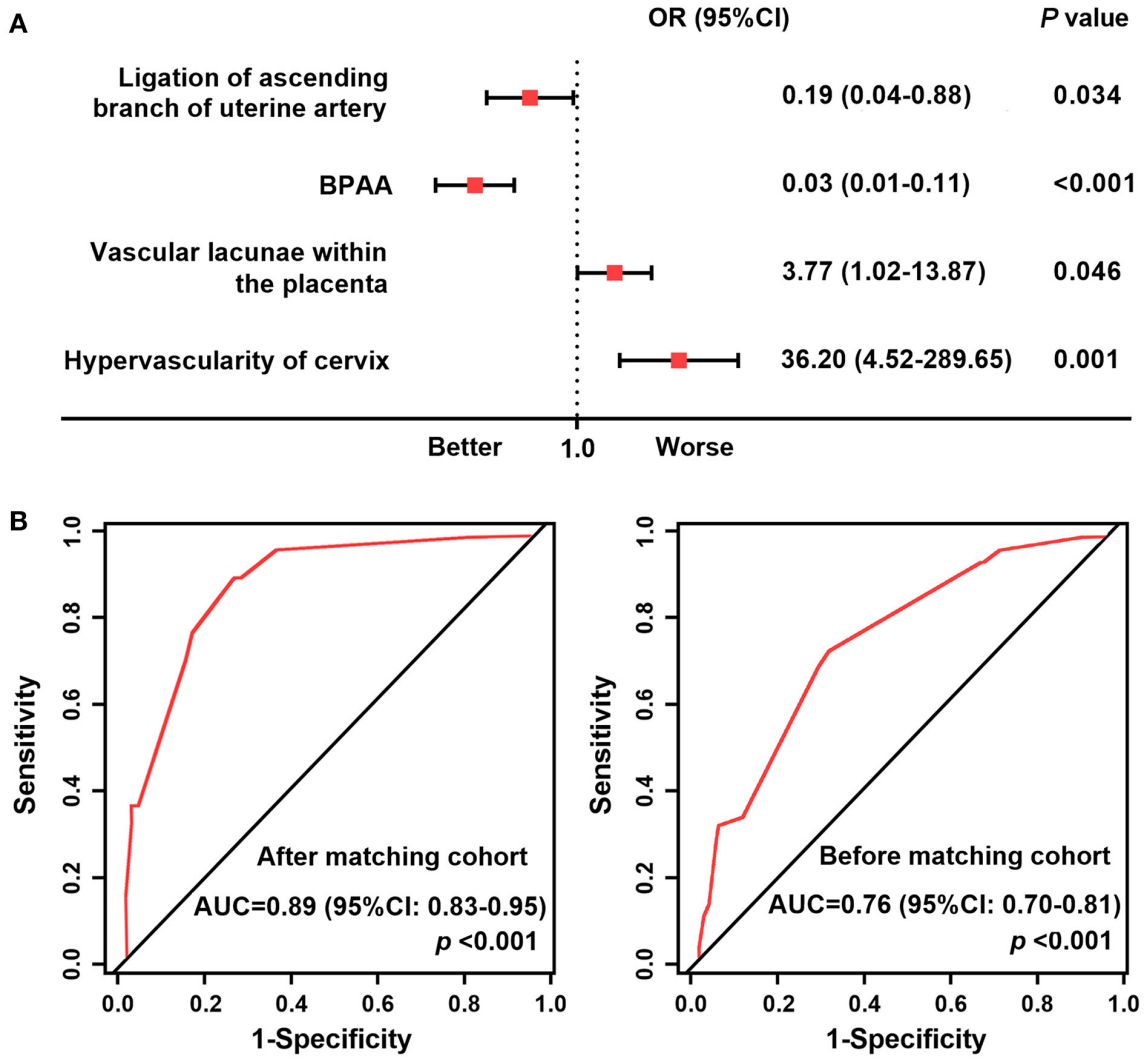
Forest plot and receiver operating characteristic curves. **(A)** Forest plot showing the result of multivariate logistic regression analysis of factors associated with a high risk of adverse maternal outcomes in the post-matched cohort. **(B)** ROC curves of the high-risk factors. AUC, area under the receiver operating characteristic curve; BPAA, balloon placement in the abdominal aorta; ROC, receiver operating characteristic.

We chose adverse maternal events as the study endpoint. Table 3 shows the results of the univariate logistic regression analysis. Following multivariate logistic regression analysis, ligation of the ascending branch of the uterine artery (*P* = 0.034), BPAA (*P* < 0.001), intraplacental vascular lacunae (*P* = 0.046), and cervical hypervascularity (*P* = 0.001) were shown to be associated with a high risk of adverse maternal events (Figure 4A). The AUC of these risk factors was 0.89 (95% CI: 0.83–0.95) in the post-matched cohort and 0.76 (95% CI 0.70–0.81) in the pre-matched cohort (Figure 4B).

**Table 3:** Univariate logistic regression analysis of risk factors associated with the adverse maternal outcome in the after propensity score matching cohort.

	**Adverse maternal outcome**	
**Characteristics**	**OR (95%CI)**	***p*** **value**
Age ≥35 years (vs. <35)	1.50 (0.68–3.29)	0.311
Gestational age <37 weeks (vs. ≥37)	1.86 (0.80–4.32)	0.148
Cesarean section >1 (vs. ≤ 1)	0.93 (0.39–2.22)	0.864
History of dilatation and curettage of uterine (vs. no)	1.63 (0.75–3.56)	0.220
HGB <100 g/L (vs. ≥100)	1.55 (0.68–3.52)	0.294
Placenta previa classification		0.778
Marginal	Reference	
Partial	–	–
Complete	0.66 (0.20–2.11)	0.478
Retroplacental myometrial thinning <1 mm (vs. ≥1)	2.31 (0.86–6.18)	0.095
Vascular lacunae within the placenta (vs. no)	2.76 (0.99–7.72)	0.052
Hypervascularity of uterine-placental margin (vs. no)	1.37 (0.55–3.40)	0.495
Irregularity of uterine-bladder interface (vs. no)	2.95 (1.25–6.98)	0.014
Hypervascularity of the uterine serosa-bladder wall interface (vs. no)	3.43 (1.52–7.74)	0.003
Hypervascularity of cervix (vs. no)	9.88 (2.08–46.86)	0.004
Emergency cesarean section (vs. no)	2.03 (0.54–7.65)	0.298
BPAA (vs. no)	0.09 (0.03–0.22)	<0.001
B-Lynch suture (vs. no)	0.60 (0.24–1.46)	0.257
Ligation of ascending branch of uterine artery (vs. no)	0.28 (0.10–0.77)	0.014
Tourniquet binding the lower uterine segment (vs. no)	0.64 (0.20–2.07)	0.458

*OR, odds ratio; HGB, hemoglobin; BPAA, balloon placement in the abdominal aorta*.

## Discussion

In this study, we used the propensity score-matching algorithm to accurately investigate the effects of BPAA on perinatal outcomes and adverse maternal events in patients with PAS and previa, who received conservative treatment. We observed that preoperative ultrasonography showed intraplacental vascular lacunae, myometrial thinning, or hypervascularity of the surrounding tissues in patients who underwent BPAA. After matching all ultrasonographic features between the groups, BPAA was associated with significantly lower rates of intraoperative blood loss, PRBC transfusion, and B-Lynch suture placement. Notably, BPAA significantly reduced the adverse maternal event rate. Therefore, BPAA plays an important role in the conservative treatment of severe PAS in patients with previa.

Conservative management of PAS includes all procedures that aim to avoid peripartum hysterectomy and the associated morbidity (8). Conservative management operations usually include additional procedures, such as interventional radiology techniques that involve temporary balloon occlusion of the iliac arteries, distal aorta, and uterine arteries (14). A meta-analysis of studies that investigated the hemorrhage control in patients with abnormal placental implantation showed that BPAA was the most effective balloon occlusion technique to control bleeding and was associated with a low complication rate (15). In our study, we observed that BPAA successfully reduced the intraoperative blood loss during cesarean delivery and could avoid hysterectomy. In our study, BPAA was performed by an interventional radiologist before the cesarean section after the umbilical cord was clamped, which is similar to the procedure reported by the previous studies (16, 17). Balloon occlusion of the aorta and consequent blockage of uterine blood flow provides more time for the obstetrician to remove the placenta and control bleeding (18).

Ultrasonography and MRI show high sensitivity and specificity for the prenatal diagnosis of placenta increta and percreta (19, 20). The findings of these imaging modalities serve as a reference for clinicians in decision-making regarding prophylactic BPAA placement before cesarean delivery. In our study, only a small number of patients who were treated on a non-emergency basis underwent preoperative MRI; therefore, owing to limited data, we could not comprehensively evaluate the severity of the patients' condition and used the results of B-scan ultrasonography to describe the specific placental findings. However, most patients in the BPAA group showed ultrasonographic features of intraplacental vascular lacunae, retroplacental myometrial thickness, as well as hypervascularity of the uterine-placental margin, and the cervix. These results were consistent with the imaging features reported by the previous studies, which indicate the severity of PAS in these patients (21, 22).

Comparison of the pre- and post-matched cohorts showed that BPAA reduced the intraoperative blood loss during cesarean delivery, which was consistent with the results of similar previous studies (12, 13, 23, 24). Furthermore, we observed that BPAA placement can lower the rate of B-Lynch suture placement. The B-Lynch suture is usually performed to control postpartum hemorrhage caused by uterine atony during cesarean delivery (25, 26). Case reports have described the uterine adhesions secondary to B-Lynch sutures; however, evidence-based data are unavailable to confirm the fertility and pregnancy outcomes in patients who underwent the B-Lynch suture for postpartum hemorrhage (27). Therefore, B-Lynch suture used during cesarean delivery warrants cautions in patients who desire fertility preservation treatment.

Previous studies have reported complications associated with balloon occlusion of arteries, including internal iliac artery thrombosis or dissection, unilateral arterial rupture necessitating thromboembolectomy, and multiple pseudoaneurysms (28–31). In our study, pulmonary embolism occurred in one patient who recovered after treatment and deep vein thrombosis in the non-interventional lower extremity in another who was treated with low-molecular-weight heparin. It is unclear whether these complications are attributable to BPAA; however, close monitoring of complications is essential during clinical treatment.

As a rule of thumb, logistic regression analysis should yield at least 10 EPVs to avoid relative bias. In our study, 46 (44.2%) patients developed adverse maternal events, and the number of EPVs was 4.4. Multivariate logistic regression analysis revealed four independent high-risk factors; therefore, the sample size of our study met the requirements.

The large cohort size and application of the propensity score-matching algorithm serve as strengths of our study. We compared perinatal outcomes of the patient cohort before and after propensity score matching, which improves the accuracy and reliability of our results. Our results showed that preoperative BPAA was associated with positive effects in the conservative treatment of severe PAS with placenta previa. Following are the limitations of our study: (A) The retrospective design of this case-control study is a drawback. (B) Patients with PAS were diagnosed during cesarean delivery by an obstetrician. Owing to the limitations of conservative management, some patients lack pathological evidence of the uterus. (C) With regard to imaging, we performed only preoperative ultrasonography in this study. Large-scale prospective studies are warranted in the future to validate our results.

## Conclusion

Conservative management using BPAA may reduce the volume of intraoperative blood loss, as well as the rate of PRBC transfusion and B-Lynch suture placement in patients with severe PAS and placenta previa, who undergo cesarean delivery with fertility preservation.

## Data Availability Statement

The raw data supporting the conclusions of this article will be made available by the authors, without undue reservation.

## Ethics Statement

This retrospective study was approved by the Ethical Committee of Qilu Hospital of Shandong University (protocol number 2019013) and obtained a waiver for informed consent. Before the analysis, the privacy of each patient was hidden. Written informed consent for participation was not required for this study in accordance with the national legislation and the institutional requirements.

## Author Contributions

RL and RC: collection and analysis, writing—initial draft, and accomplishing the final version. QW, YX, YZ, GT, and QL: data analysis. YZ, YX, and YM: surgeons of the patients. YM: study concept, design, supervision, and revision of the article. All authors contributed to the article and approved the submitted version.

## Funding

This work was supported by the Key Research and Development Program of Shandong Province (Grant Number 2019GSF108048).

## Conflict of Interest

The authors declare that the research was conducted in the absence of any commercial or financial relationships that could be construed as a potential conflict of interest.

## Publisher's Note

All claims expressed in this article are solely those of the authors and do not necessarily represent those of their affiliated organizations, or those of the publisher, the editors and the reviewers. Any product that may be evaluated in this article, or claim that may be made by its manufacturer, is not guaranteed or endorsed by the publisher.
